# REVISITING ELECTRONIC FRAILTY INDEX BY ADDING A LAB-BASED MARKER OF NUTRITION: A HEART FAILURE COHORT

**DOI:** 10.1093/geroni/igac059.2717

**Published:** 2022-12-20

**Authors:** Seulgi Kim, Ariela Orkaby, Catherine Park, Molly Horstman, Salim Virani, Orna Intrator, Aanand Naik, Javad Razjouyan

**Affiliations:** Baylor College of Medicine, Houston, Texas, United States; VA Boston, Boston, Massachusetts, United States; Veterans Affairs, Houston, Texas, United States; Veterans Affairs, Houston, Texas, United States; Baylor College of Medicine, Houston, Texas, United States; Veterans Health Administration, Rochester, New York, United States; Veterans Affairs, Houston, Texas, United States; Veterans Affairs, Houston, Texas, United States

## Abstract

Malnutrition is associated with worse prognosis and increased risk for adverse outcomes in patients hospitalized with heart failure (HF). The objective is to assess the utility of adding the Prognostic Nutritional Index (PNI), a validated measure of nutritional status, to the Veterans Health Administration frailty index (VA-FI) in predicting time to death in patients hospitalized with HF. We conducted a retrospective cohort study of veterans age ≥50 years hospitalized with HF as their primary diagnosis. PNI was calculated using lab values in the year prior to hospitalization with the following equation: 10 x serum albumin (g/dL)+0.005 x total lymphocyte count (mm3). VA-FI identified five groups: robust (≤0.1), prefrail (0.1–0.2), frail (0.2–0.3), moderately frail (0.3–0.4), and severely frail (>0.4). PNI was added to VA-FI (VA-FI-Nutrition) using the same cutoffs. We identified changes in frailty status using VA-FI versus VA-FI-Nutrition by summarizing the count by each class and reported the hazard ratio (HR) for all-cause mortality in each VA-FI category based on the new VA-FI-Nutrition groups. VA-FI-Nutrition identified patients within each VA-FI class that belong to the next frailty strata: robust (20.2%), prefrail (18.3%), frail (16.7%) and moderately frail (16.7%). We observed higher mortality rates among those whose frailty class changed based on VA-FI-Nutrition compared to VA-FI: robust (HR, 1.65, 95%CI:1.38, 1.97), prefrail (HR, 1.52, 95%CI: 1.41, 1.65), frail (HR, 1.42, 95%CI: 1.33, 1.52), and moderately frail (HR, 1.33, 95%CI: 1.24, 1.43). Adding PNI to VA-FI provides a more accurate mortality assessment and may be utilized to triage high-risk patients.

